# Characterization of a rare case of Ullrich congenital muscular dystrophy due to truncating mutations within the COL6A1 gene C-Terminal domain: a case report

**DOI:** 10.1186/1471-2350-14-59

**Published:** 2013-06-05

**Authors:** Elena Martoni, Stefania Petrini, Cecilia Trabanelli, Patrizia Sabatelli, Anna Urciuolo, Rita Selvatici, Adele D'Amico, Sofia Falzarano, Enrico Bertini, Paolo Bonaldo, Alessandra Ferlini, Francesca Gualandi

**Affiliations:** 1 Department of Medical Science, Section of Medical Genetics, University of Ferrara, Ferrara, Italy; 2 Unit of Muscular and Neurodegenerative Disorders, Department of Neuroscience, Bambino Gesu’ Hospital, Rome, Italy; 3 IGM-CNR, Unit of Bologna c/o IOR, Bologna, Italy; 4 Department of Histology, Microbiology and Medical Biotechnologies, University of Padua, Padua, Italy

**Keywords:** Ullrich congenital dystrophy, Collagen VI, C-terminal truncating mutations

## Abstract

**Background:**

Mutations within the C-terminal region of the COL6A1 gene are only detected in Ullrich/Bethlem patients on extremely rare occasions.

**Case presentation:**

Herein we report two Brazilian brothers with a classic Ullrich phenotype and compound heterozygous for two truncating mutations in COL6A1 gene, expected to result in the loss of the α1(VI) chain C2 subdomain. Despite the reduction in COL6A1 RNA level due to nonsense RNA decay, three truncated alpha1 (VI) chains were produced as protein variants encoded by different out-of-frame transcripts. Collagen VI matrix was severely decreased and intracellular protein retention evident.

**Conclusion:**

The altered deposition of the fibronectin network highlighted abnormal interactions of the mutated collagen VI, lacking the α1(VI) C2 domain, within the extracellular matrix, focusing further studies on the possible role played by collagen VI in fibronectin deposition and organization.

## Background

Collagen type VI is a widespread heterotrimeric extracellular matrix glycoprotein; it is mainly present in the stroma, but it also forms a microfibrillar network in close association with the basement membrane of most tissues [1].

Its subunits, the α1(VI), α2(VI), and α3(VI) chains, are encoded by the *COL6A1*, *COL6A2* (chr.21q22.3) [2,3] and *COL6A3* (chr.2q37) [3] genes respectively and share a central triple-helix (TH) domain with repeating Gly-Xaa-Yaa sequences flanked by N- and C-globular domains [4-6].

Formation of collagen VI is a complex multi-step process: inside the cells, the equimolar association of the three subunits to form a triple-helical monomer is followed by assembly into disulphide-bonded anti-parallel dimers, which then align to form tetramers, also stabilized by disulphide bonds. Outside the cell, the tetramers, the secreted form of collagen VI, associate end-to-end through overlapping N-terminal globular domains, thereby forming double-beaded microfibrils [1].

Mutations in the *COL6A1*, *COL6A2*, and *COL6A3* genes cause collagen VI-related myopathies, a group of allelic disorders exhibiting a variable combination of muscle wasting and weakness, joint contractures, distal laxity, and respiratory compromise [7-9].

Ullrich congenital muscular dystrophy (UCMD, OMIM #254090), caused by both inherited recessive and dominant *de novo* COL6 mutations, is the most severe of these disorders. In UCMD patients, collagen VI is typically reduced or absent in the muscle and in cultured skin fibroblasts [10-12]. The majority of COL6 gene mutations reported in UCMD patients result in premature termination codons [7,8; Leiden Muscular Dystrophy pages http://www.dmd.nl/col6a1, http://www.dmd.nl/col6a2, and http://www.dmd.nl/col6a3]. In addition, missense mutations substituting glycine in the TH Gly-Xaa-Yaa motif are frequently reported [7,8,13], as well as splicing mutations leading to in-frame exon deletions [7,8,14].

Although clear mutational hot spots have not been identified, exon 10 of COL6A1, exon 26 of COL6A2 and intron 16 of COL6A3 seems to be preferentially mutated [8] and the topographical distribution of mutations along the different protein domains differs between the chains. In the α2(VI) chain, mutations have been described affecting N-terminal, TH and C-terminal domains to a similar degree. In contrast, mutations in α1(VI) and α3(VI) chains are almost exclusively located in the TH and N-terminal domains, with just few in frame deletions and missense changes affecting the C-terminal regions have been described and in general mutations in these C-domains being very rare [8; Leiden Muscular Dystrophy pages http://www.dmd.nl/col6a1, http://www.dmd.nl/col6a2 and http://www.dmd.nl/col6a3]. Indeed, no truncating mutations have been described in this domain of the α3(VI) chain, and only one case of UCMD carrying a homozygous truncating mutation within the a1(VI) chain C-terminus has been reported [15].

In this study we characterize the clinical, transcriptional, immunohistochemical and biochemical features of a rare example of truncating mutations within the C-terminal domain of the COL6A1 gene, detected in two Brazilian brothers with UCMD.

## Case presentation

The two Brazilian brothers both have a clinical diagnosis of UCMD; they were born from non-related parents, neither of whom reported a family history of neuromuscular diseases. The eldest patient was unable to walk autonomously until he was 3 years of age, whereupon he consistently showed a waddling gait, a severe difficulty in climbing stairs and rising from the floor, and a total inability to run. At the age of 5 he lost the ability to walk. Clinical examination at 9 years evidenced: severe muscle weakness, predominantly involving proximal muscles, marked hyperlaxity of the skin and distal joints and contractures of the knees and elbows. Respiratory function and serum CK were normal, and no significant scoliosis was reported.

**Figure 1:**
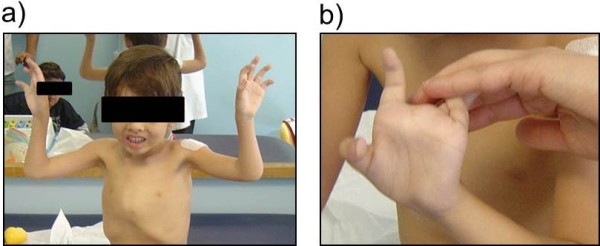
Clinical presentation of the youngest sib: a) The patient is unable to lift his arms against the force of gravity; b) detail on hyperlaxity of fingers.

The youngest sib has a similar clinical presentation but, differently from the brother, he was never able to walk. At the age of examination (5 years), he was completely unable to lift his arms or legs against the force of gravity. He also displayed skin and joint hyperlaxity (Figure 1) and mild contractures of the hips and knees. Neither scoliosis nor respiratory problems were evident.

## Methods

### DNA and RNA analyses

The study was approved by the local ethics committee (Comitato Etico Della Provincia di Ferrara). Genomic sequencing of COL6 genes was performed, as previously described, on both patients [16].

Total RNA was isolated from confluent skin fibroblasts from the eldest patient by RNeasy Kit (QIAGEN). RT-PCR was performed as previously described [9], using amplification primers within exons 26, 31, 32 and 35 of the *COL6A1* gene. RT-PCR products were sequenced either directly or subsequent to cloning into pCRII -TOPO vector (Invitrogen).

Real-Time PCR was performed on cDNA from fibroblasts, using TaqMan expression assays for the three *COL6A* genes (*COL6A1*: Hs01095581_g1 Ex 16-17; *COL6A2*: Hs00964570_g1 ex 13–14; *COL6A3*: Hs00915102_m1 Ex 23–24 from Applied Biosystems) and for actin as a reference gene (*ACTB* Endogenous Control), as previously described [9].

### Immunofluorescence and confocal microscopy analysis on skin fibroblasts

Dermal fibroblast cultures were obtained from skin biopsy explants from the eldest patient as previously described [17]. Confluent cells were incubated with an anti-collagen VI antibody (MAB1944, Millipore, Temecula, CA), followed by AlexaFluor 488/anti-mouse IgG (Invitrogen, Molecular Probes, Carlsbad, CA). In double-staining experiments, samples were incubated with a goat anti-fibronectin antibody (Santa Cruz), followed by AlexaFluor 555/anti-goat IgG (Molecular Probes). The other samples were formaldehyde fixed, permeabilized with 0.15% Triton X-100, and incubated with MAB1944 antibody. Nuclei were counterstained with Hoechst (Molecular Probes) or DRAQ5 (Biostatus, UK). Slides were mounted with ProLong anti-fade reagent (Molecular Probes).

Confocal imaging was performed using Olympus Fluoview FV1000 confocal IX81 microscope, using a 20× objective lens (0.75 N.A.). Colocalization analysis and image processing were carried out as reported [17].

### Electron microscopy of secreted collagen VI

After 10 days’ treatment with 0.25 M ascorbic acid (to allow collagen VI secretion), cultured fibroblasts from the eldest sibling were incubated with a monoclonal anti-collagen VI antibody (Chemicon), diluted 1:25 with Dulbecco’s modified Eagle medium, and 5 nm colloidal gold-labelled IgG (Amersham). Rotary shadowing electron microscopy was performed as previously described [18]. Replicas were observed under a Phillips EM400 electron microscope operating at 100Kv.

### Immunoblotting of skin fibroblast-derived collagen VI

Fibroblasts were grown in the presence or absence of L-ascorbic acid; the medium and cell layer were collected from confluent cultures, and Western blot was performed under reducing conditions as previously described [9]. Antibodies used were: a polyclonal antiserum recognizing all collagen VI chains (Fitzgerald, 7CR-CR009X, lot x 11040530) and a polyclonal antiserum against human α3(VI) chains (donated by Raimund Wagener, University of Cologne). An antibody against GAPDH (Millipore, MAB437) was used as a loading control. Analysis of the media has been performed using comassie blu stained gel.

## Results

### DNA and RNA analyses

DNA analysis of the UCMD brothers revealed a compound heterozygosis for recessive COL6A1 mutations: an insertion within exon 33 (c.2331 ins/dup GCCT) of maternal origin, and a G>C substitution affecting the intron 32 donor splice site (c. 2148+1; NM_001848) inherited from the father.

**Figure 2:**
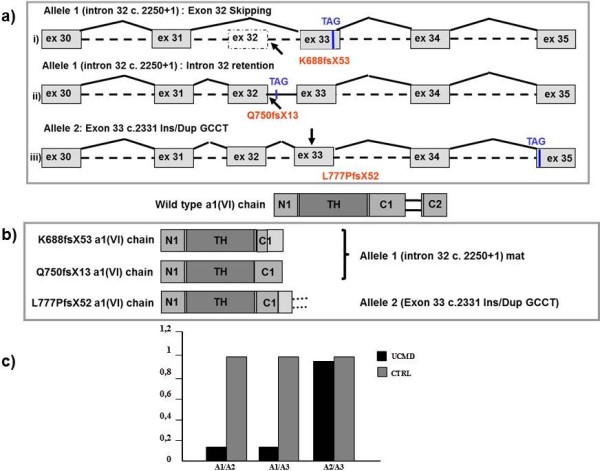
**Schematic rappresentation of aberrant transcripts and protein products, and relative quantification of the mutated COL6A1 messenger. a)** Schematic representation of the out-of-frame COL6A1 transcripts deriving from the two alleles carrying the mutated intron 32 c.2250+1 (allele 1) and exon 33 c.2331 Ins/Dup GCCT (allele 2). The in-frame stop-codons recognized as an effect of the frame-shifting mutations are indicated. **b)** Schematic representations of the predicted protein products arising from the identified mutations. All truncated a1 (VI) chains lack the C2 subdomain. **c)** Relative quantification of the mutated COL6A1 messenger with respect to COL6A2 and COL6A3 wild-type transcripts. COL6A1 RNA was reduced to 15% of COL6A2 and COL6A3 mRNA levels, while the COL6A2/COL6A3 ratio (1:1) was similar to that displayed by an unaffected control.

Transcriptional analysis on RNA from cultured fibroblasts from the eldest patient, revealed multiple COL6A1 out-of-frame transcripts resulting from different pathological events: exon-32 skipping (K688fsX52), intron-32 retention (Q750fsX13) and exon-33 heterozygous ins/dup GCCT (L777PfsX52) (Figure 2a and Additional file 1: Figure S1).

The in-frame stop-codons resulting from the identified mutations fell either within the C1 subdomain or within the cysteine-rich connecting region. Consequently, the overall predicted effects consisted of the production of truncated α1(VI) chains selectively lacking the C2 subdomain of the C-terminal region (Figure 2b).

Real-Time PCR showed a strong reduction in the aberrant COL6A1 transcripts with respect to non-mutated COL6A2 and COL6A3 mRNA. In contrast, the ratio between the levels of COL6A2 and COL6A3 mRNA was similar to that observed in an unaffected control (Figure 2c).

### Immunofluorescence and electron microscopy

**Figure 3:**
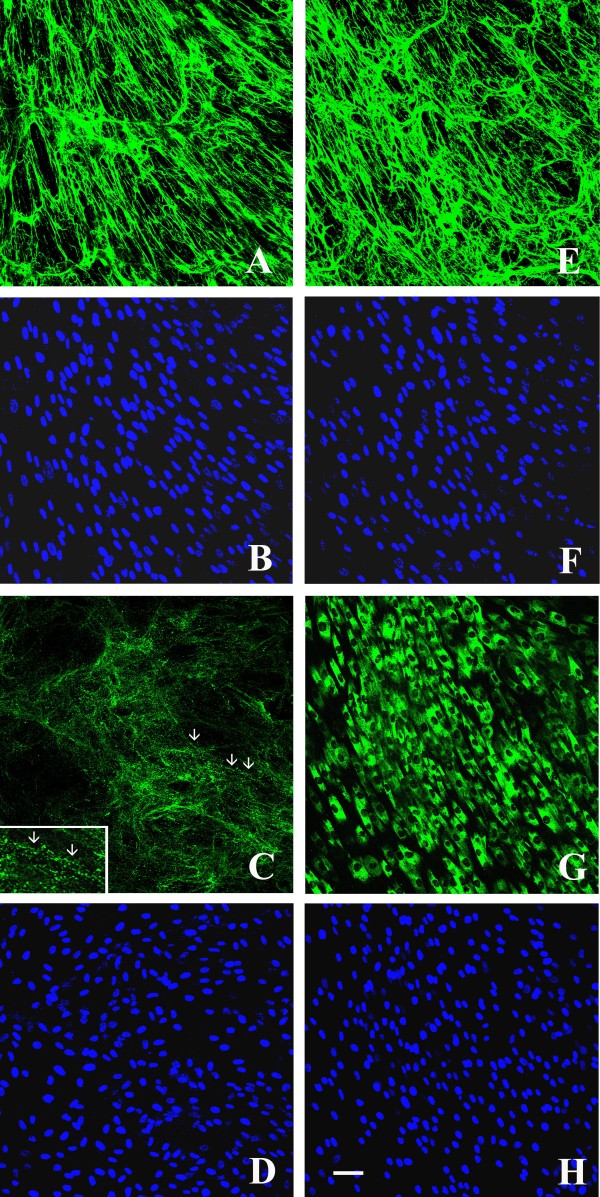
**Confocal laser microscopy of the collagen VI matrix in control and UCMD-confluent dermal fibroblasts after 4-days of L-ascorbic acid treatment.***In vivo* staining of the collagen VI network in control fibroblasts detected a normal quantity and a parallel alignment of collagen VI microfibrils **(a)**, whereas UCMD cells secreted a decreased amount of collagen VI **(c)**, and microfibrils showed a punctuate and discontinuous pattern of deposition (arrows, inset). In fixed and permeabilized samples, collagen VI immunofluorescence detected a significant intracellular retention in the majority of UCMD fibroblasts **(g)**, whereas control cells exhibited a normal collagen VI deposition **(e)**. Cell density was checked by counterstaining with Hoechst **(b, f, d, h)**. Scale bar: 50 μm.

**Figure 4:**
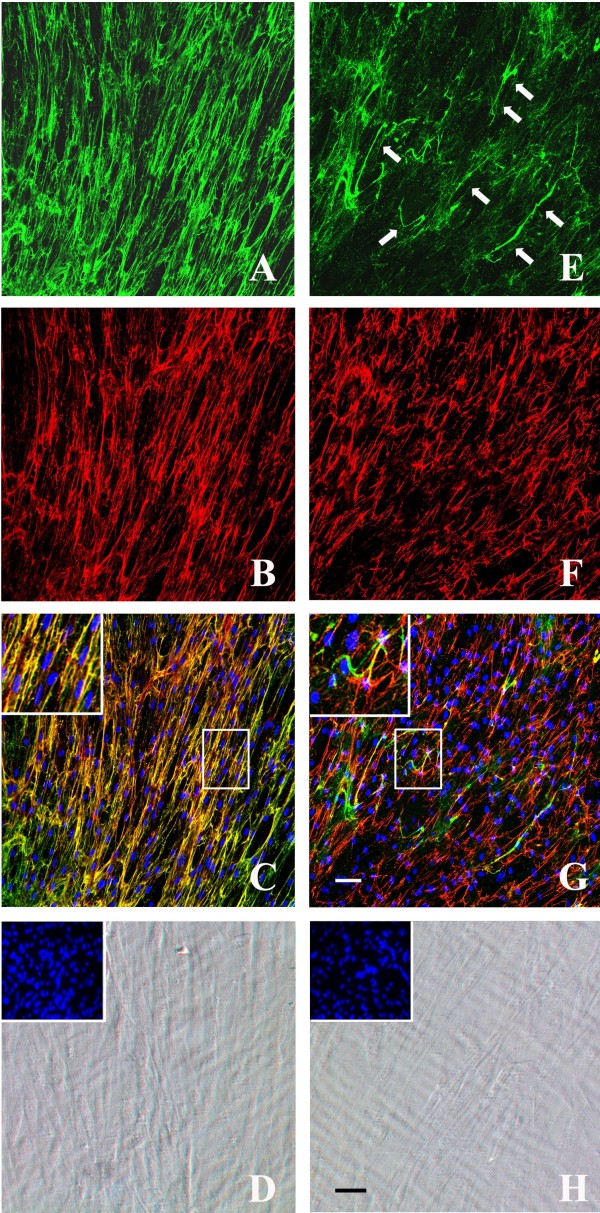
**Double immunolabelling with collagen VI and fibronectin antibodies.** Double immunolabelling with collagen VI (*green*) and fibronectin (*red*) antibodies of the extracellular matrix secreted by cultured dermal fibroblasts from control **(a-c)** and UCMD patient **(e-g)** after 10 days of ascorbate treatment. Phase contrast images of control **(d)** and UCMD **(h)** fibroblasts showed the state of cell confluence (nuclei were stained with Hoechst, in insets). In control cells, collagen VI **(a)** and fibronectin **(b)** networks showed a similar distribution pattern and colocalized in several points, as revealed by their merge signal (*yellow*, high magnification in the inset). UCMD fibroblasts secreted a decreased amount of collagen VI matrix **(e)** with rare bundles of short microfilaments (**e**, arrows). UCMD cells produced an altered fibronectin network with short and irregularly aligned fibrils **(f)**, and the colocalization with the collagen VI matrix was significantly impaired (**g**, high magnification in the inset). Nuclei were stained with DRAQ5 (pseudocolored in *blue* in **c** and **g**). Scale bar: 50 μm.

After 4 days of ascorbate treatment, the total amount of collagen VI matrix in cultured UCMD fibroblasts was significantly reduced (Figure 3c), and microfibrils displayed a punctuate and discontinuous appearance (Figure 3c, inset). Permeabilization of cellular samples revealed an abnormal intracellular retention of collagen VI in the majority of UCMD fibroblasts (Figure 3g). After 10 days of ascorbate treatment, no effect on the discontinuous and punctuate distribution of collagen VI network was observed in the UCMD matrix, although some groupings of microfibrils into short microfilaments were detectable (Figure 4e, arrows). Additionally, fibrils from the fibronectin matrix were short and less copious (Figure 4f), and scarce colocalization between fibronectin fibrils and the rare bundles of collagen VI microfilaments was evident (Figure 4g inset).

**Figure 5:**
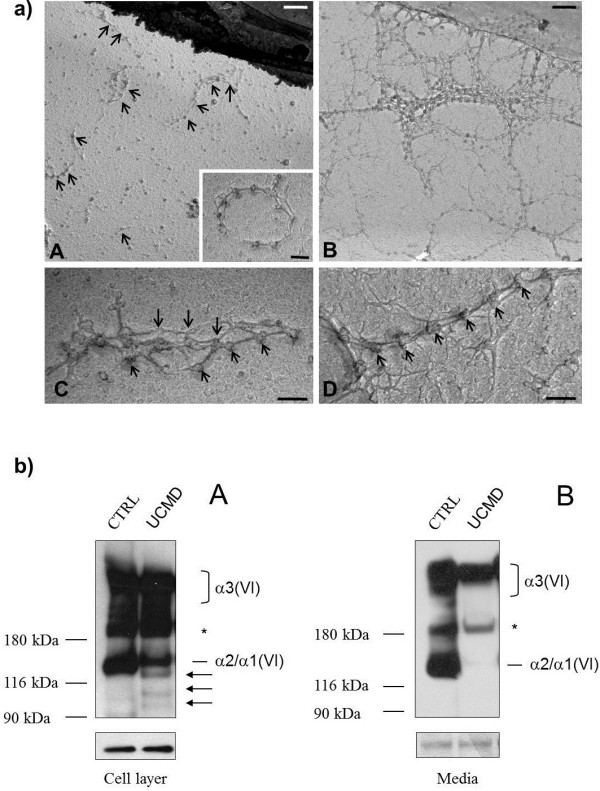
**Electron microscopy and western blot of Collagen VI. a)** Electron microscopy of collagen VI secreted by fibroblasts from the UCMD patient (**A**, **C**) and controls (**B**, **D**). Collagen VI microfibrils from the patient (**A**, arrows) appear randomly deposited in the extracellular matrix, and unable to interconnect and develop regular webs with respect to normal fibroblasts (**B**). A ring structure constituted by a single microfilament is shown in the inset in panel **A**. Collagen VI fibrils from the patient are disorganized, and poor alignment was detected in the globular domains of parallel microfibrils (**C**, arrows). In contrast, fibrils from normal fibroblasts show the typical regular beaded organization and parallel association of globular domains (**D**, arrows). Scale bars: 400 nm in A-B, and 100 nm in **C**-**D**. **b)** Western blot of collagen VI in cell layer and medium of the UCMD patient and a healthy donor. Samples derived from skin fibroblasts of the patient (UCMD) and a healthy donor (CTRL), were separated by SDS-PAGE onto 3-8% polyacrylamide under reducing conditions. The molecular weight markers (kDa) are indicated on the left. The arrows point to the three truncated α1 (VI) products, while the asterisk refers to a non-specific band. The loading control in the cell layer samples was performed using a GAPDH antibody. The expected migration of collagen VI chains are indicated on the right.

After 10 days of L-ascorbic acid treatment, examination by transmission electron microscopy revealed short collagen VI microfibrils which appeared unable to develop regular webs (Figure 5a A, B). Ring structures constituted by single collagen VI microfilaments were also detected (Figure 5a A inset). Short collagen VI fibrils, constituted by parallel microfibrils, were occasionally found, and the globular domains of adjacent microfibrils were not regularly aligned as in control fibroblasts (Figure 5a C, D).

### Western blot analysis

In the UCMD cell layer, besides bands corresponding to α3(VI) and α2(VI) chains, we identified three bands migrating at about 120 kDa, 100 kDa and 90 kDa (Figure 5b A). The estimated molecular weight of these three bands correspond to the expected migration for the protein variants coded by the different out-of-frame transcripts produced by patients cells. Interestingly, none of these truncated protein variants were detected in the conditioned medium of patient cells (Figure 5b, B). The amount of collagen VI detected in the cell layer from the patient was similar to control (Figure 5b, A). In contrast, the patient medium was found to contain significantly less collagen VI than that of the unaffected donor (Figure 5b, B).

## Discussion

Mutations occurring in the C-terminal region of the COL6A1 gene are exceptionally rare. The few missense changes reported in this region exhibit the allele frequencies of common polymorphisms [7,8] Leiden Muscular Dystrophy pages [http://www.dmd.nl/], and only a single case of truncating mutation has been reported to date ([15] Patient P2). This UCMD patient, described by Giusti et al. carries an homozygous nonsense mutation within COL6A1 exon 31 (Tyr659X), causing an out-of-frame skipping of the mutated exon in 15% of the transcripts. Collagen VI was absent in the muscle, and Western blot analysis of cultured cells failed to detect the α1(VI) chain in either cells or medium.

We investigated the transcriptional, immunohistochemical and biochemical behaviour of two truncating mutations within the C-terminal region of the COL6A1 gene occurring in compound heterozygosis in two UCMD brothers born from healthy parents.

Transcriptional analysis revealed the occurrence of multiple aberrant out-of-frame COL6A1 transcripts which, despite the dramatic reduction of COL6A1 RNA due to nonsense RNA decay, give rise to truncated protein products, predicted to lack the α1(VI) C2 subdomain, as detectable by Western blot analysis. This behaviour was reflected in the severe reduction in collagen VI matrix seen in cultured fibroblasts, and in the abnormal intracellular retention observed.

A variety of studies have highlighted the importance of C-terminal interactions in the alpha1(VI), alpha2(VI) and alpha3(VI) chains in chain selection, association and triple helix formation [19]. In the extracellular matrix of our patient cultures, a minute amount of collagen VI could be detected, as indicated by immunofluorescence and electron microscopy, suggesting that the absence of alpha1(VI) C2 domain does not entirely impair the association of mutant protein with alpha2 and alpha3 chains. However, our finding that the secreted tetramers do not form extensive networks indicates that the alpha1(VI) C2 domain is critical for tetramer alignment and interactions with other components of the extracellular matrix.

Although the precise protein interactions in the organization of collagen VI in the extracellular matrix remain unclear, fibronectin is known to interact with the alpha1 (VI) chain [20]. Fibronectin-collagen VI interaction involves the globular domain of collagen VI, which contains the amino and carboxyl-terminal domains, and fibronectin three-dimensional arrangement is modulated by collagen VI deposition [21]. Interestingly, fibronectin organization was found to be altered in the extracellular matrix of the analysed patient indicating that the α1(VI) C2 domain might be involved in fibronectin deposition and organization. Previous studies suggested that a defective secretion and deposition of collagen VI may impair the organization of fibronectin fibrils [21] in the extracellular matrix of cultured fibroblasts, as well as smaller amount of fibronectin protein may be detected in the UCMD matrix respecting to control cells [17].

## Conclusions

Considering the high frequency of mutations affecting the COL6A2 C-domain, the rarity of UCMD/BM mutations involving the COL6A1 C-terminal region is intriguing, especially in view of the structural and evolutionary similarities between COL6A1 and COL6A2 genes, and thus far remains unexplained [22]. Based on their location in the same region of chromosome 21, it has been suggested that the two genes derive from a duplication of a primordial collagen VI gene; this hypothesis would explain the highly similar exon structure of their triple helical domains and the similar structure of α1(VI) and α2 (VI) chains [23]. However, these genes differ in the region encoding the globular domains [22], and conservation in the C-terminus is limited to the region containing the cysteine residues essential for correct collagen assembly.

This divergence could reflect disparate, still unclarified, functional roles of the α1 (VI) and α2 (VI) C2 domains in the context of collagen VI interactions within the extracellular matrix, also possibly justifying the different contribution of C-terminal mutations of the two proteins to collagen VI-related phenotypes. The altered deposition of the fibronectin network highlighted abnormal interactions of the mutated collagen VI, lacking the α1(VI) C2 domain, within the extracellular matrix, focusing further studies on the possible role played by collagen VI in fibronectin deposition and organization.

### Consent

Written informed consents were obtained from the patients for publication of this Case report and any accompanying images. A copy of the written consent is available (Dr. E Bertini – Dr. A. D’Amico) for review by the Series Editor of this journal.

## Abbreviations

BM: Bethlem myopathy; ColVI: Collagen type VI; COL6A: Gene(s) encoding the alpha chain(s) of collagen VI; CK: Creatine kinase; PTC: Premature termination codon; RT-PCR: Real-Time PCR; TH: Triple Helical domain; UCMD: Ullrich congenital muscular dystrophy.

## Competing interests

The authors declare that they have no competing interests.

## Authors’ contributions

ME performed RNA studies and wrote the manuscript; PS performed immunohistochemical analysis; TC performed genomic sequencing; SP performed electron microscopy studies; UA performed Western Blot analysis; SR critically revised the manuscript; DA and BE performed the clinical assessment of patients; FS performed cells cultures; BP supervised Western Blot studies; FA conceived the work and critically revised the manuscript; GF supervised the studies and critically revised the manuscript. All authors read and approved the final manuscript.

## Pre-publication history

The pre-publication history for this paper can be accessed here:

http://www.biomedcentral.com/1471-2350/14/59/prepub

## Supplementary Material

Additional file 1: Figure S1 RT-PCR with primers 31F-35R shows 4 distinct bands with different molecular weight (left). All bands were characterized by sequencing: the upper corresponds to a PCR artefact. Details on sequencing of each band is shown or the right. Click here for file
