# Supplementary figures and images for: Characterization of a rare case of Ullrich congenital muscular dystrophy due to truncating mutations within the COL6A1 gene C-Terminal domain: a case report
Source: BMC Med Genet. 2013 Jun 5;14:59. doi: 10.1186/1471-2350-14-59 (PMC3681647; doi:10.1186/1471-2350-14-59)

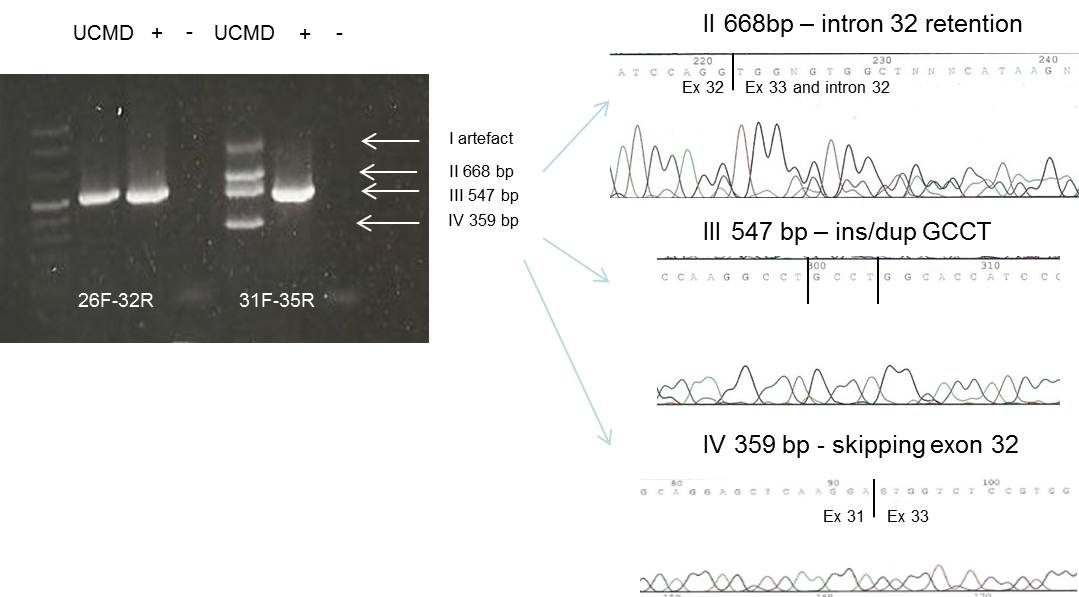

Supplement: Additional file 1: Figure S1 — RT-PCR with primers 31F-35R shows 4 distinct bands with different molecular weight (left). All bands were characterized by sequencing: the upper corresponds to a PCR artefact. Details on sequencing of each band is shown or the right. [file 1471-2350-14-59-S1.jpeg]
